# Preparation and Characterization of Chloroprene Latexes Modified with Vinyl-POSS

**DOI:** 10.3390/polym16040462

**Published:** 2024-02-07

**Authors:** Junhua Chen, Zhenxian Wu, Qingwei Wang, Chuanghui Yang, Jinlian Chen, He Zhang, Yinping Wu, Dong-Yu Zhu, Xiangying Hao

**Affiliations:** 1 School of Environmental and Chemical Engineering, Zhaoqing University, Zhaoqing 526061, China; cehjchen@yeah.net (J.C.); wuzhenxian2023@163.com (Z.W.); wangqingwei0721@163.com (Q.W.);; 2 Guangdong Provincial Key Laboratory of Environmental Health and Land Resource, College of Environmental and Chemical Engineering, Zhaoqing University, Zhaoqing 526061, China; 3 School of Chemical Engineering and Light Industry, Guangdong University of Technology, Guangzhou 510006, China; zdy16@gdut.edu.cn

**Keywords:** vinyl-POSS, chloroprene latexes, tensile strength, thermal stability

## Abstract

Water-based chloroprene latex is a solvent-free, environmentally friendly adhesive. Currently, its market demand is growing rapidly. However, there are problems such as a lack of heat resistance and poor mechanical properties, which limit its application. The introduction of vinyl-POSS (OVS) into the resin structure can effectively improve the thermal stability of chloroprene adhesives. In this paper, modified waterborne chloroprene latex was prepared by copolymerization of methyl methacrylate and OVS with chloroprene latex. The results showed that vinyl-POSS was successfully grafted onto the main chain of the waterborne chloroprene latex, and the modified waterborne chloroprene latex had good storage stability. With the increase in vinyl-POSS, the tensile strength of the chloroprene latex firstly increased and then decreased, the tensile property (peel strength of 20.2 kgf) was maintained well at a high temperature (100 °C), and the thermal stability of the chloroprene latex was improved. When the addition amount was 4%, the comprehensive mechanical properties were their best. This study provides a new idea for the construction of a new and efficient waterborne chloroprene latex system and provides more fields for the practical application of waterborne chloroprene latex. This newly developed vinyl-POSS modified chloroprene latex has great application potential for use in home furniture, bags, and seat cushions.

## 1. Introduction

Chloroprene latex, a type of water-based emulsion chloroprene rubber adhesive, is composed of polychloroprene micelles and various types of emulsifiers [1,2,3,4]. This versatile adhesive is widely utilized in bonding fabrics, leather, wood, plastics, glass, and other materials, playing a crucial role in various industries worldwide [5,6,7,8]. The environmental friendliness, safety, non-toxicity, non-flammability, and affordability of water-based chloroprene latex are notable advantages [9,10,11,12,13]. However, it also possesses certain drawbacks, such as slow drying, limited substrate compatibility, low initial viscosity, poor resistance to extreme temperatures, susceptibility to freezing, poor storage stability, and color change over time. Considering these limitations, the modification of waterborne chloroprene latex adhesives is often necessary to enhance their overall performance [14,15]. Various industries rely on the development of chloroprene adhesives to meet their specific bonding needs. In recent years, significant efforts have been made to improve the formulation of chloroprene latex adhesives, addressing their inherent limitations, and expanding their application range. These efforts have resulted in the development of modified chloroprene latex adhesives with improved drying times, enhanced substrate compatibility, increased initial viscosity, and better resistance to environmental factors, such as heat and cold. Furthermore, advancements in storage stability and color retention have contributed to the broader use of chloroprene latex adhesives in diverse industrial settings. As a result, the market for modified chloroprene latex adhesives continues to grow, offering superior performance and versatility for various bonding applications.

There are several common methods for modifying chloroprene latex, including blending modification, graft modification, and copolymerization modification [16,17,18,19,20,21]. Blending modification is prone to phase separation problems, making it less suitable for certain applications. Graft modification, on the other hand, is a more useful method of polymer modification as it is easy to control various reaction parameters [22,23]. In the past, Kai Zhang et al. grafted methyl methacrylate and styrene onto polychloroprene rubber using emulsion polymerization. By incorporating the bulking agent made using this method, they achieved better mechanical properties of the contact rubber compared to simple blends [14].

Polyhedral oligosiloxanes (POSS) are small molecules that have cage-like three-dimensional structures at the nanoscale. The general formula for POSS is (RSiO_1.5_)_n_ [24,25,26]. The core of POSS is made up of inert inorganic materials, such as organosilicon and oxygen (SiO_1.5_). When incorporated into a polymer matrix, POSS enhances the mechanical properties and thermal stability of the polymer [27,28,29,30]. The substituents (R) attached to the silicon atoms situated at the corners of the cage can be classified as hydrogen, reactive, or inert organic groups [31,32]. These organic groups promote specific interactions and compatibility between POSS and the polymer or monomer [33,34]. By utilizing traditional chemical transformations, it is possible to replace one or more of the substituents with functional groups. These multifunctional groups, such as methacrylates, styrenes, epoxides, alcohols, and phenols, facilitate the introduction of POSS into a polymer chain or network through grafting or polymerization [35,36,37,38]. The addition of nanosized inorganic particles is an effective approach to enhance the properties of polymers while maintaining their low density and high ductility [39]. Moreover, when applied to emulsion polymerization, POSS does not suffer from the problem of alkoxy hydrolysis polycondensation that general organosilicon monomers encounter. Furthermore, POSS can form connections with multiple organic groups due to its high reactivity [40,41]. The vinyl group in OVS (organosilicon vinyl ether) allows for the conversion of OVS into valuable products through various reactions [42,43]. The objective of this study was to modify waterborne chloroprene latex using functional silicone macro-monomers with a nanoscale three-dimensional structure derived from OVS. The effects of these modifications on the stability, latex particle size, heat resistance, and T-peel strength were investigated. The modified chloroprene rubber can be widely used in home furniture, bags, seat cushions for aircraft, cars, high-speed trains, trains, ships, and other modes of transportation. Additionally, it can be employed in the bonding of leather, sponge, plastics, and wood [40,44,45].

## 2. Materials and Methods

### 2.1. Materials

Industrial-grade chloroprene latex (CRL) was purchased from Shanghai Costron Polymers Co., Ltd., Shanghai, China. The methyl methacrylate (MMA), sodium dodecyl biphenyl ether sulfonate (DSB), and vinyl-POSS (95%, OVS) used in this study were all analytical reagent grade and were purchased from Shanghai Maclin Biochemical Technology Co., Ltd., Shanghai, China. Tert-butyl hydroperoxide (TBHP) and tetraethylenepentamine (TEPA) were all analytical grade and were purchased from Shanghai Aladdin Biochemical Technology Co., Ltd., Shanghai, China. Benzene propylene emulsion was industrial reagent grade and was purchased from Shandong Haoshun Chemical Co., Ltd. (Jinan, China). Deionized water is homemade in the laboratory. All the other reagents underwent further purification before use.

### 2.2. Preparation of Modified Aqueous Chloroprene Latex

**Scheme 1:**
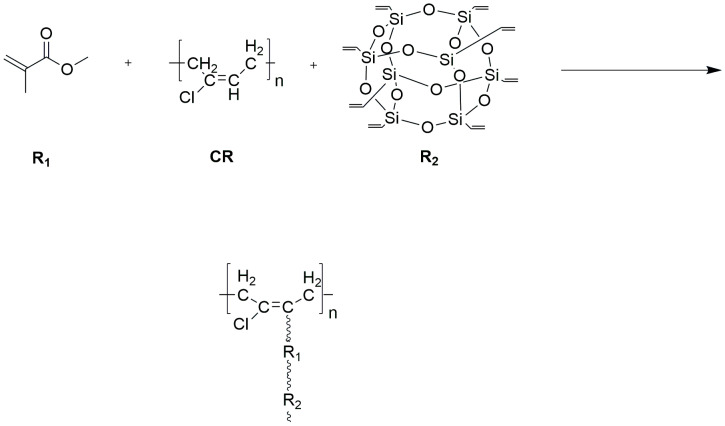
OVS modified aqueous chloroprene synthetic approaches.

The grafting reaction was conducted using the seed emulsion polymerization method displayed in Scheme 1. A 250 mL three-necked round bottom flask equipped with a thermostatically heated magnetic stirrer, peristaltic pump, condenser pipe, and thermometer was used. In this flask, 25 g of CRL and DSB (1% of the total monomer weight) was added. The reaction system was then diluted with deionized water to achieve a solid content of 30% while stirring with a thermostatically heated magnetic stirrer at 50 °C. An amount of 8.25 g of mixed monomer (MMA and OVS) was added dropwise at a uniform rate over 100 min. The OVS component accounted for 0–5% of the total weight ratio of monomer and polymer. During this process, a mixture of TBHP (TBHP/TEPA = 1:1, 0.5% of the dry weight of CRL) was added, alongside the dropwise addition of aqueous TEPA, maintaining a constant temperature for 3.5–4 h. Finally, the emulsion was allowed to cool to room temperature to obtain the OVS-modified aqueous chloroprene latex.

### 2.3. Characterization

**Scheme 2:**
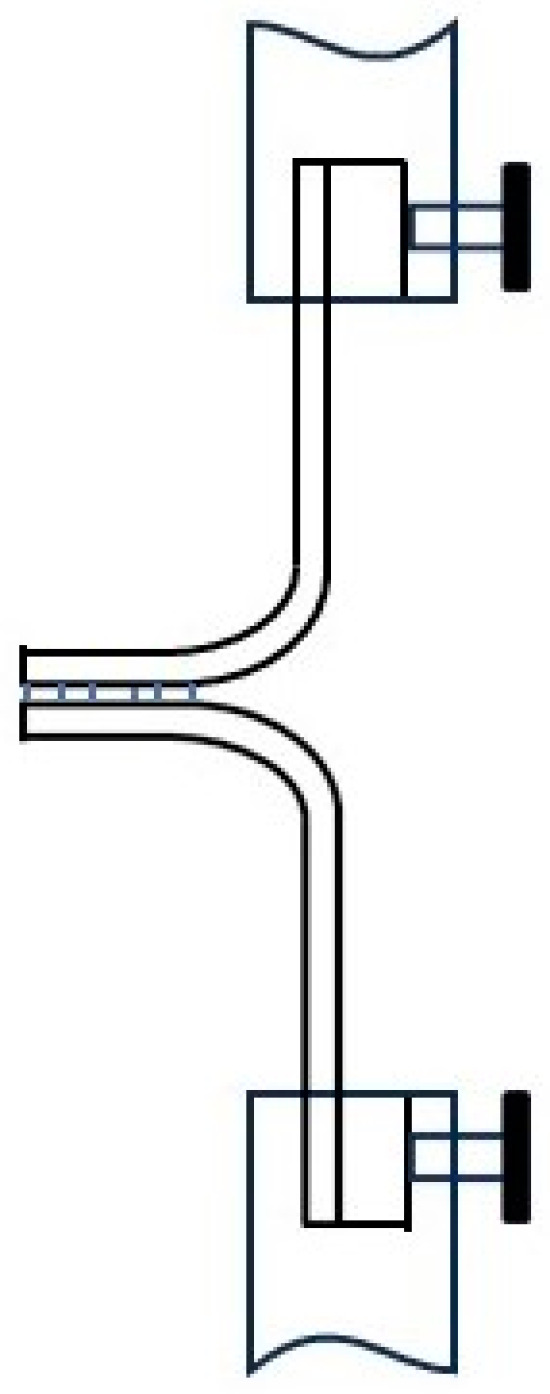
Schematic diagram of T-peeling test.

Fourier transform infrared spectroscopy (FT-IR) analysis of the samples was obtained in the range of 400–4000 cm^−1^ using a Nicolet IS50-Nicolet Contin FT-IR spectrometer (Thermo Fisher Scientific Inc., Waltham, MA, USA). The viscosity of the samples was measured using a DVS+ viscometer according to the standard GB/T 2794-1995 [46]. The TG and DTG curves were obtained by heating the samples from 40~600 °C at a scanning rate of 10 °C/min under a nitrogen atmosphere using a TG209F1 thermogravimetric analyzer (NETZSCH-Gerätebau GmbH., Selb, Germany). The samples were thermally analyzed to obtain the glass transition temperature by DSC 214 with a temperature scanning range of 80–120 °C, a heating rate of 10 °C/min and a nitrogen atmosphere. The T-peeling strength of the sample was measured using a temperature-controlled tensile strength machine according to the standard of GB/T 2790-1995 [47] displayed in Scheme 2.

To test the stability of the emulsion dilution, 2 mL of emulsion was diluted in 10 mL of water. Then, after a week, the mixture was observed to see if any phenomena such as precipitation or delamination occurred. The emulsion samples were placed in 50 mL clear vials and left at room temperature (25 °C, 50% humidity) for 24 h. To test the stability of the emulsion at room temperature during the measurement period, a visual method of observation was used. In an oven set to a constant temperature of 50 °C, the emulsion samples were put. To ascertain the emulsion’s stability at high temperatures, the emulsion was visually examined during the measurement period to see if flocculation or emulsion breakup occurred.

The emulsion samples and phenylpropylene emulsion were mixed together before being uniformly applied to a piece of sponge. A second piece of sponge was then aligned and bonded, and after waiting for it to dry at room temperature and being placed into the oven for an hour at a high temperature, the heat resistance of the latex was assessed by testing its tensile properties both before and after exposure to high temperature. The synthesized vinyl-POSS modified neoprene emulsion samples were co-mingled with the phenylpropylene emulsion and coated uniformly on the paper skins, and the coated paper skins (15 cm × 3.5 cm) were aligned and glued together with another paper skin, rolled with a rolling machine and pressed until 48 h. In accordance with the GB/T 2909 standard [48], the T-peel strength of the sample emulsion was tested using an Al-7000S temperature-controlled tensile machine (High Speed Rail Testing Instruments Ltd., Taipei City, Taiwan, China) at a testing temperature of 40 °C and with a cross-head speed of 100 mm/min.

The mechanism of polymerization kinetics was to use a linear regression equation to find the slope *(dx/dt*) of the monomer conversion (*x*)–time (*t*) curve in the constant velocity phase, and then the rate of polymerization $R_{p}=\frac{dX}{dt}\times \left[M_{0}\right]$ was calculated according to the formula, where the formula [*M*_0_] = the amount of substance of the monomer (mol)/volume of water in the formulation (L).

## 3. Results and Discussion

**Figure 1:**
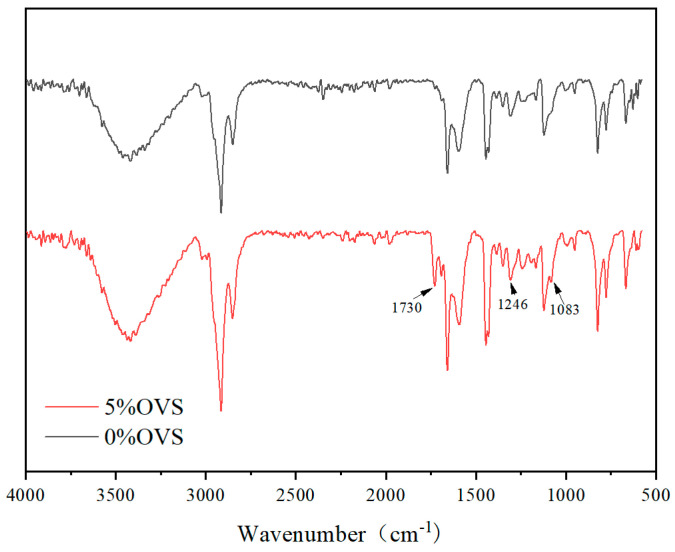
Infrared spectra of CRL and 5% OVS-CRL.

As shown in the infrared spectra of 0% OVS-CRL and 5% OVS-CRL in Figure 1, the 5% OVS waterborne chloroprene latex, in addition to the typical characteristic peaks, has a strong and sharp ester group C=O telescoping vibration peak at 1730 cm^−1^ and a C-O telescoping vibration peak at 1246 cm^−1^. The peaks at 1083 cm^−1^ represent the stretching vibrations of the siloxane network (Si-O-Si). This indicates that methyl methacrylate and vinyl-POSS have been successfully grafted onto the chain of waterborne chloroprene latex.

The effect of the addition of OVS on the viscosity, solid content and conversion rate of chloroprene latexes was investigated with the same amount of chloroprene, emulsifier, initiator and reducing agent added, the content of fixed MMA and other conditions being the same, and the results are shown in Table S1. As can be seen from Table S1, as the content of the monomer OVS increases, the viscosity decreases, with the first conversion increasing and then decreasing. As the conversion decreases, the latex particles decrease, the water component increases, the total surface area of the system particles decreases, making the interaction and resistance to movement between the latex particles weaken, and the Brownian motion of the latex particles becomes easier, so the viscosity decreases.

**Figure 2:**
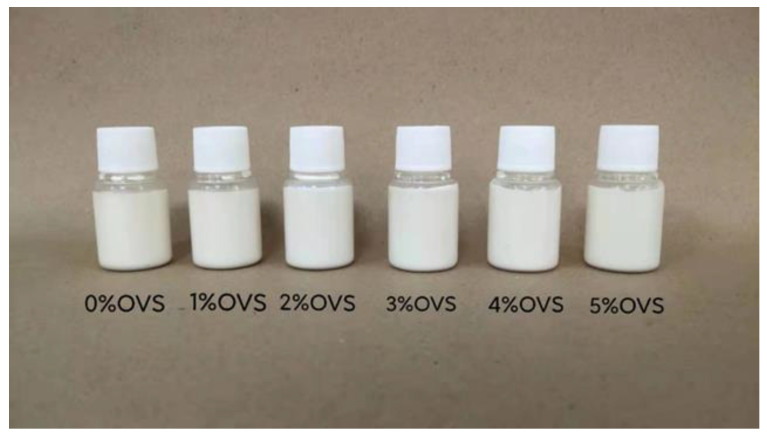
The appearance state of the OVS modified emulsion after 90 days of placement.

Table S2 demonstrates the stability of OVS modified chloroprene emulsions. As the content of monomer OVS increased, the samples with 0% to 3% OVS content had a good appearance without gelation, and the emulsions reacting at 4% and 5% OVS content produced a small amount of gelation. The emulsion has good dilution stability, room temperature and high temperature storage stability. Among them, Figure 2 shows the appearance state of OVS-modified waterborne chloroprene emulsion after 90 days of placement. The modified emulsion was light yellow in color with no obvious delamination and precipitation.

**Figure 3:**
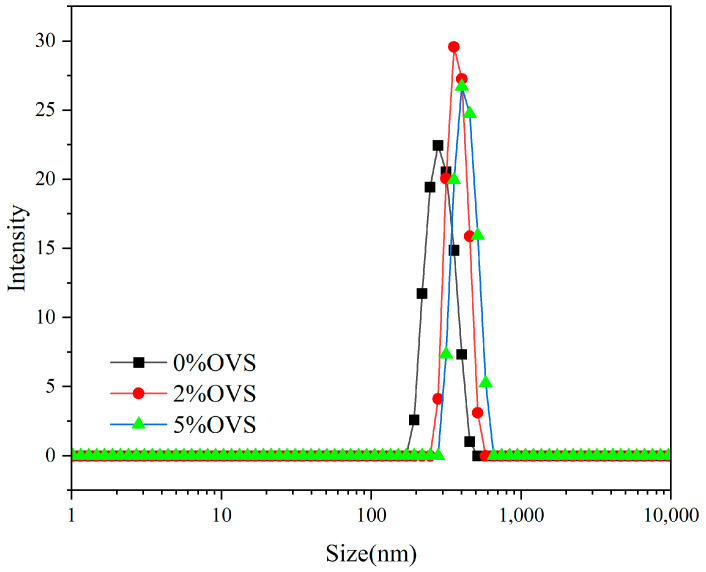
Particle size distribution of modified waterborne chloroprene latex.

Figure 3 shows the particle size distribution of modified aqueous chloroprene latexes with 0%, 2% and 5% OVS content, respectively.

The graph reveals that the particle sizes of the modified chloroprene latexes with 0%, 2%, and 5% OVS are all single-peaked. The average particle size of the 0% OVS-modified waterborne chloroprene latex is 275.9 nm, while that of the 2% OVS-modified latex is 355.6 nm. The particle size of the 5% OVS-modified latex is the largest, at 401.5 nm. As the OVS content in the monomer increases, the average particle size gradually increases. This observation suggests that the modified chloroprene latex has been successfully grafted with MMA and OVS, leading to larger latex particles. Furthermore, there is no phase separation in the modified latex, indicating successful grafting and a more homogeneous emulsion. The increase in particle size can be attributed to the presence of OVS, which acts as a stabilizer and allows for more-controlled particle growth. The larger particle size may also be attributed to the fact that OVS contains vinyl groups that can undergo polymerization reactions, resulting in a more cross-linked and stable latex network. The grafting of MMA and OVS onto the chloroprene rubber backbone not only alters the particle size but also affects other properties of the latex, such as its mechanical properties and thermal stability.

**Figure 4:**
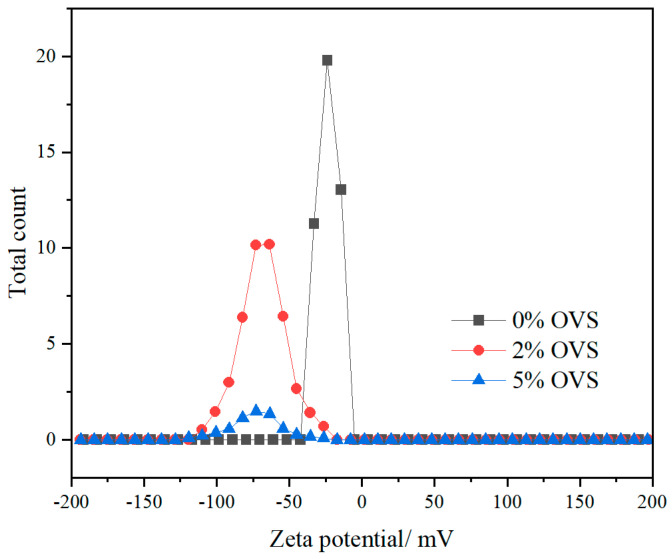
Potential distribution of modified waterborne chloroprene latex.

Figure 4 presents the potential distribution of the modified waterborne chloroprene latexes containing 0%, 2%, and 5% OVS content, respectively. It can be observed that the potentials of the modified chloroprene latexes with different OVS contents are single-peaked. The potential of the 0% OVS-modified latex is −23.3 mV, while that of the 2% OVS-modified latex is −68.3 mV. The potential of the 5% OVS-modified latex is the lowest, at −71.7 mV. As the OVS content in the monomer increases, the potential of the modified waterborne chloroprene latex gradually decreases. This trend suggests that most of the CRL particles are compatible with OVS and MMA, leading to a more stable colloidal dispersion system. The potential distribution curve is a useful tool for understanding the stability and compatibility of the latex particles. A single-peaked curve indicates that the particles are relatively uniform in size and that the colloidal dispersion system is stable. The shift in potential as the OVS content increases can be attributed to changes in the surface charge and interactions between the particles. The compatibility between CRL particles and OVS/MMA is crucial for the mechanical and physical properties of the final product. A high degree of compatibility ensures that the particles remain well-dispersed and do not aggregate, leading to a more uniform final material. This uniformity in turn affects various properties such as tensile strength, tear resistance, and hardness, among others. Therefore, understanding the potential distribution and compatibility of the latex particles is essential for optimizing the final product’s performance.

**Figure 5:**
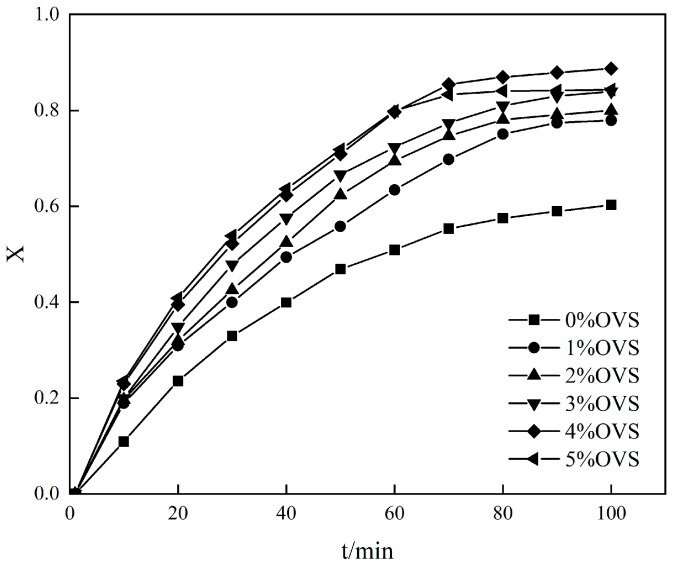
Effect of OVS concentration on polymerization kinetics.

The concentration of aqueous chloroprene latex was fixed, DSB was added at 1% of the mass of the mixed monomer, TBHP-TEPA was added at 0.5% of the dry weight of CRL, the solid content of the system was controlled at 30%, the polymerization temperature was 50 °C, the concentration of MMA was fixed, and the effect of the variation of vinyl POSS concentration on the polymerization rate was investigated; the results are shown in Figure 5.

As Figure 5 demonstrates, the polymerization rate of the reaction increased as the concentration of OVS increased. Initially, the polymerization rate gradually increased with the polymerization time, indicating growth in the number of polymer chains. This was followed by a period of constant rate polymerization, where the rate remained relatively constant. However, as the polymerization proceeded further, the polymerization rate gradually decreased. The increase in the polymerization rate with increasing OVS concentration can be attributed to the enhanced initiation and propagation reactions that occur due to the presence of vinyl groups on POSS. The vinyl groups act as reactive centers, facilitating the addition of monomer units to the growing polymer chains. This leads to an increase in the number of active polymer chains and ultimately a higher polymerization rate. The observed decrease in polymerization rate towards the end of the reaction can be explained by factors such as the depletion of monomer, a decrease in the availability of reactive sites on the growing polymer chains, or chain termination reactions that become more prevalent as the polymerization progresses. These factors result in a decrease in the number of active polymer chains and a corresponding reduction in the polymerization rate.

**Figure 6:**
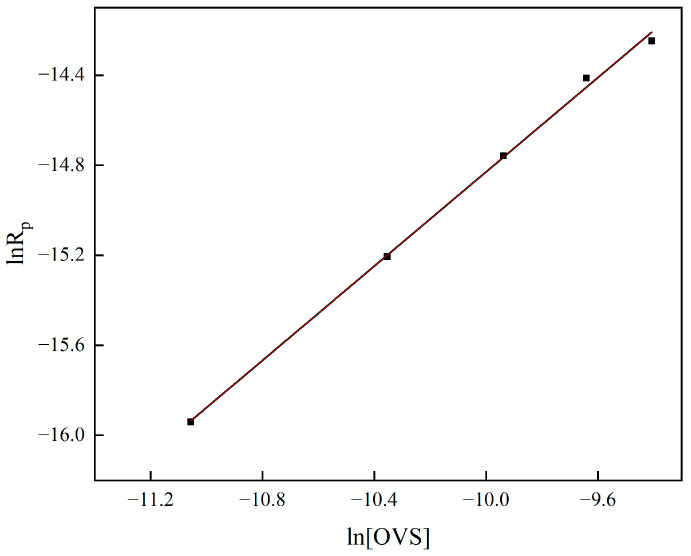
Relationship between lnRp and ln[OVS].

Figure 6 shows a plot of lnRp versus ln[OVS] with a linear regression with a linear slope of 1.05 and a correlation coefficient of 0.99912, which yields the equation for the relationship between the reaction rate and monomer concentration: Rp∝[OVS]^1.05^, with a correlation coefficient of 0.99912, deviating from the classical kinetic model of emulsion polymerization (Rp∝[OVS]^1^) with a monomer reaction order greater than 1. It can be correlated that at low conversions, the relationship between reaction rate and reactant concentration is R_p_ = K[E]^0.15^[I]^0.30^[OVS]^1.05^ (K is a constant). The rate equation Rp = k_p_(2kd/kt1)^0.5^[M][I_2_]^0.5^ derived from the reaction mechanism is basically correct for the radical reaction mechanism of graft polymerization, but there are deviations in the order of reaction because the actual experimental procedure deviates from the assumptions made during the theoretical derivation.

**Figure 7:**
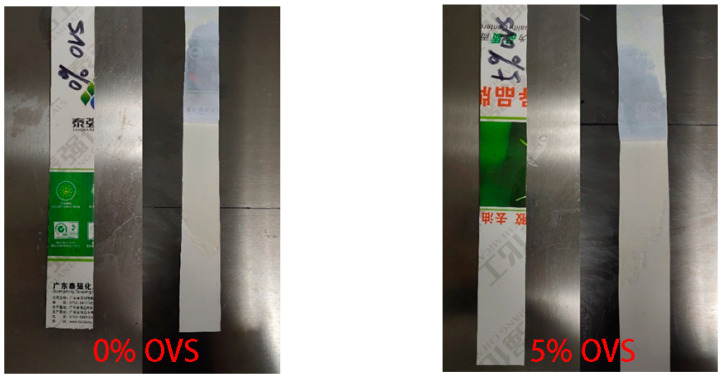
The surface of the adhesive sample before and after stretching.

Figure 7 shows a before and after photograph of 0% and 5% OVS modified waterborne chloroprene latex tested for stretching, with no tearing of the substrate after stretching, which indicates that all have good peeling properties. When interface failure occurs between the adhesive and the substrate, the peel strength of the sample modified with 5% OVS is approximately 0.30 kgf/mm.

**Figure 8:**
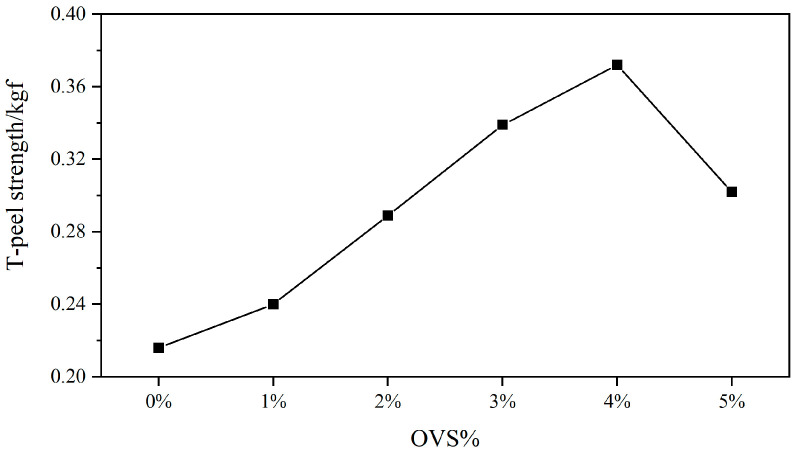
Effect of OVS concentration on T-peel strength.

Figure 8 illustrates that the T-peel strength of modified aqueous chloroprene latex increased and then decreased as the addition of OVS increased, which was due to the increased concentration of OVS, which increased the vinyl involved in the silicone hydrogen addition reaction in the system and increased the crosslinking density of the system, making the crosslinking point dense. The cross-linking reaction makes the molecular chain of chloroprene latex grow, and the flexible molecular chain can disperse the stress well under the action of external force; meanwhile, the cross-linking reaction enhances the force between the monomer and chloroprene latex, which improves the mechanical properties of chloroprene latex. Meanwhile, the cross-linking reaction enhances the force between the monomer and chloroprene latex, which improves the mechanical properties of chloroprene latex; moreover, due to the unique inorganic hollow cage skeleton of OVS, the chloroprene latex enhances the rigidity. When the content of OVS reaches 5%, the monomer is not uniformly dispersed in the system due to the excessive addition of OVS, resulting in agglomeration, which makes the internal bonding of the chloroprene rubber latex poorer, reduces the tensile strength, and, consequently, reduces the mechanical properties. Table S3 shows the mechanical properties of OVS-modified neoprene rubber in detail. From the table, the tensile strength and elongation at break gradually increase with the increase in OVS content. When the OVS content is 5%, the tensile strength is 1.45 MPa and the elongation at break is 523.3%. Compared with the reported mechanical properties of neoprene–montmorillonite nanocomposite, the OVS-modified neoprene rubber improved significantly [49].

**Figure 9:**
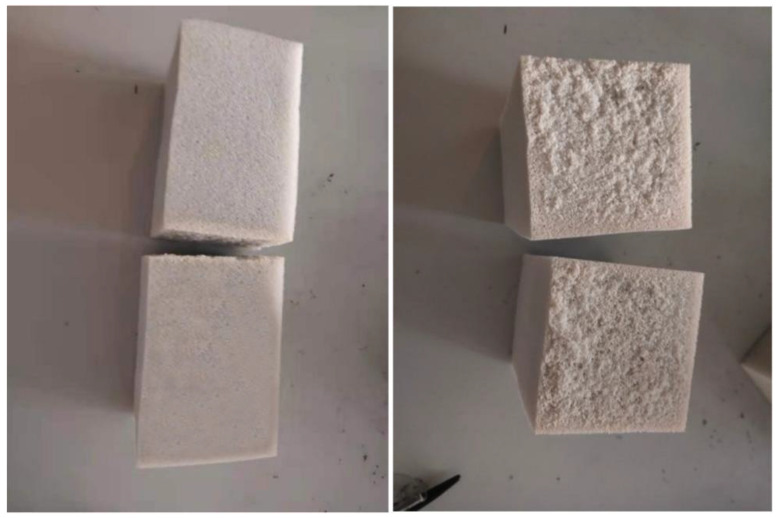
Tensile fracture cross-sectional morphology of unmodified neoprene latex specimens (fracture at the sample bond).

**Figure 10:**
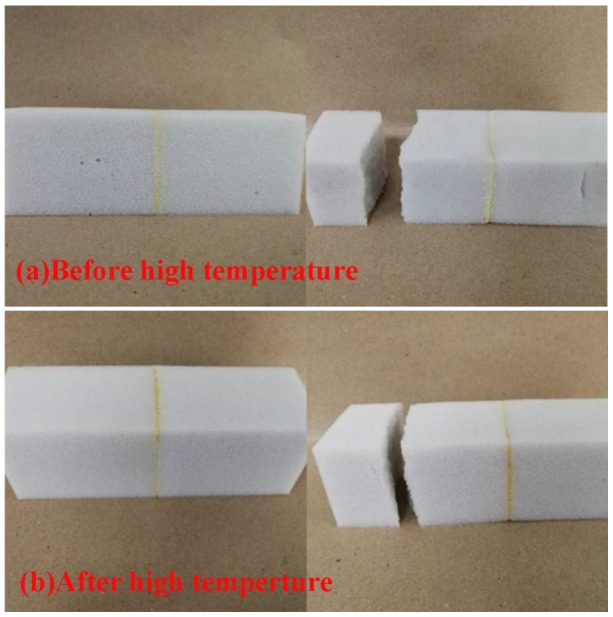
Tensile fracture of modified chloroprene latex samples at high temperatures (modified samples did not break from the bond before and after 100 °C).

Figure 9 reveals that the unmodified neoprene latex samples undergo rupture and debonding at the bonding site after stretching. In contrast, Figure 10 demonstrates that the OVS-modified neoprene latex sample strip exhibits breakage upon stretching. Notably, there is no debonding observed at the bonding site after stretching at room temperature. However, there is material breakdown present at other locations, indicating that the bonding performance is still satisfactory at 100 °C. These observations suggest that the addition of OVS enhances the bonding properties of the neoprene latex. The improved bonding performance can be attributed to the presence of vinyl groups on POSS, which facilitate stronger interactions between the polymer chains and the substrate. This leads to a more robust material that is better able to withstand stretching without debonding. Furthermore, the fact that the bonding performance remains good at elevated temperatures highlights the potential of OVS-modified neoprene latexes for applications where thermal stability is essential, such as in high-temperature seals or gaskets.

After the tensile property test, the tensile fracture of sponge bonded with 4% OVS modified chloroprene latex was 16.81 kgf before high temperature treatment and 20.20 kgf after high temperature treatment. The peel strength of OVS modified chloroprene latex after high temperature treatment was larger than that before high temperature treatment, and both of them broke the material, indicating that the addition of OVS improved the heat resistance of water-based chloroprene latex. 

**Figure 11:**
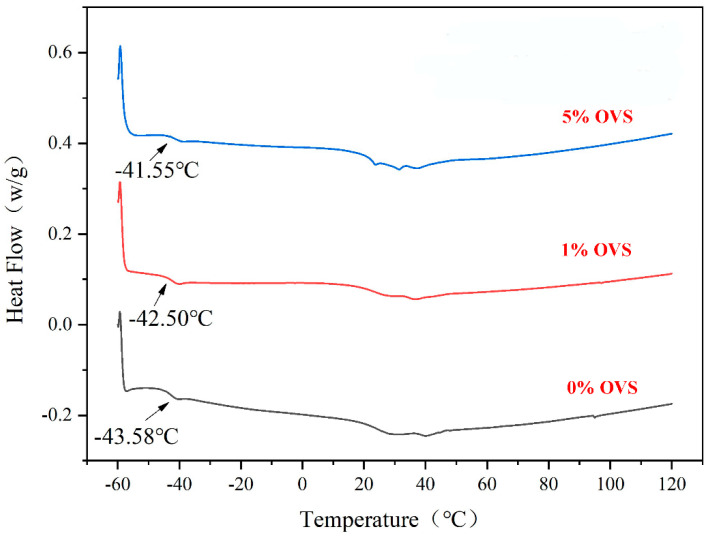
DSC curves of modified waterborne chloroprene latex with 0% OVS, 1% OVS and 5% OVS.

Figure 11 displays the DSC curves of the modified waterborne chloroprene latexes containing 0%, 1%, and 5% OVS content, respectively. As observed in the figure, the glass transition temperature (T_g_) of the 0% OVS-modified waterborne neoprene latex adhesive is −43.58 °C. For the 1% OVS-modified adhesive, the T_g_ is −42.50 °C, and for the 5% OVS-modified adhesive, the Tg is −41.55 °C. These results indicate that the addition of OVS increases the T_g_ of the adhesive. The T_g_ is influenced by various factors, including the mobility of both the main chain and side groups of the macromolecules. The observed increase in T_g_ suggests that the addition of OVS impairs the movement of chloroprene rubber macromolecular chain segments. This is likely due to the organosilicone resin present in OVS, which acts to hinder the flexibility of the molecular chain segments and increase their rigidity. With increasing OVS content, there is a corresponding increase in cross-linking within the molecular chain. This cross-linking leads to an increase in chain rigidity and a corresponding rise in the glass transition temperature. The more OVS that was added, the more severe the cross-linking becomes, leading to a further increase in chain rigidity and T_g_.

**Figure 12:**
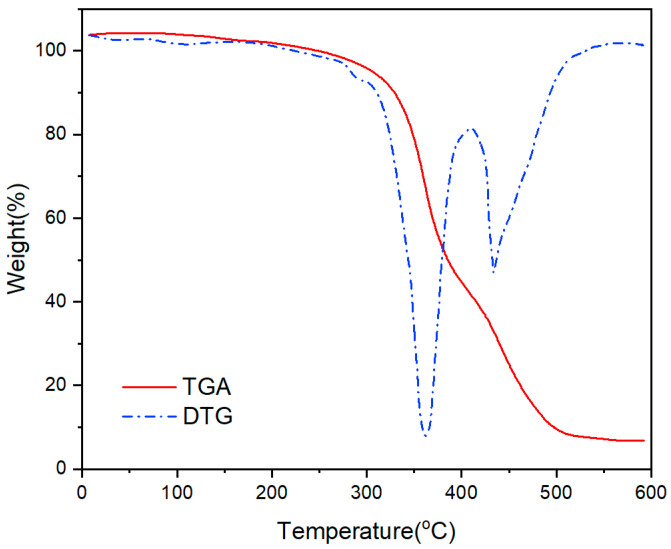
TGA and DTG curves of 0% OVS modified waterborne chloroprene latex.

**Figure 13:**
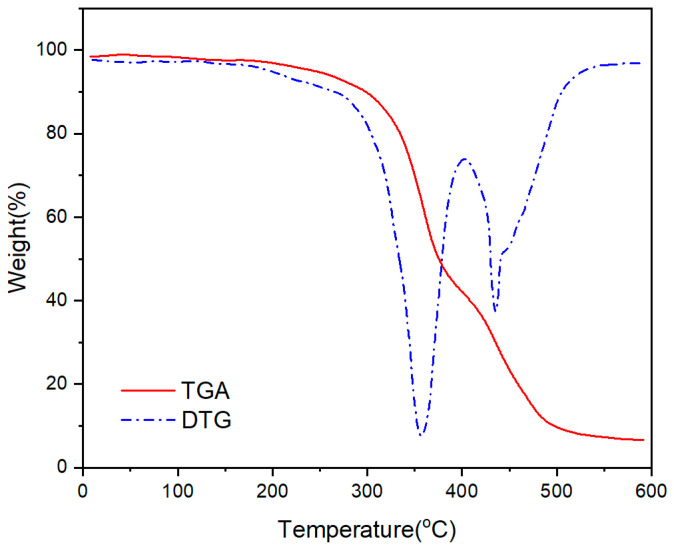
TGA and DTG curves of 5% OVS modified waterborne chloroprene latex.

Figure 12 and Figure 13 show the TG curves of the modified waterborne chloroprene latexes with 0% and 5% OVS content, respectively, while the temperature of maximum mass loss rate (T_max_) and char yield (Y_c_) are summarized in Table S4. 

The weight loss of all coatings was approximately 1 wt% at approximately 100 °C, probably due to the small amounts of volatile solvents and water contained in the system. The first decomposition stage occurs at 250~420 °C, mainly due to C-C and C-H degradation of the main chain. The second decomposition stage occurs in the temperature range of 350~500 °C, which may be related to the ester bonds in the structure and the C-Cl degradation structure. We found that the lowest T_max1_ was 372.6 °C and the highest T_max2_ was 450.4 °C when the OVS content was 5%, which was mainly due to the grafting of vinyl-POSS onto the chloroprene latex backbone, which increased the initial decomposition temperature of the backbone polymer but decreased the final decomposition temperature. The increase in initial decomposition temperature was attributed to the vinyl-POSS branched grafting increasing the interchain interaction force and decreasing the degree of crystallinity, thus increasing the heat resistance. The addition of vinyl-POSS improved the thermal stability of the neoprene adhesives. This is due to the formation of a three-dimensional network of the system when vinyl-POSS is added, which reduces the flexibility of the polymer chains and delays the final decomposition temperature. This is also confirmed by Table S4. The residual carbon content based on OVS-0% and OVS-5% at 600 °C is 18.58 wt% and 21.64 wt%, respectively. This is mainly due to the introduction of siloxanes with alkoxyl groups into the main chain of the neoprene polymers, resulting in a three-dimensional network crosslinked structure. Therefore, the addition of OVS improves the thermal stability of the bonding layer. Compared with the chemically modified neoprene rubber with cashew phenol grafting reported in the literature, the OVS-modified neoprene studied in this paper has a significantly higher residual carbon rate at 600 °C because of the structure of the silicone-oxygen network in the OVS, so it has a higher thermal stability and a higher T_g_ [50].

## 4. Conclusions

(1) Waterborne chloroprene latex modified with vinyl-POSS was prepared by emulsion polymerization using a redox initiator (TBHP/TEPA 0.5% of dry weight), an emulsifier (DSB 1% of total monomer) at a polymerization temperature of 50 °C, with a solid content of 30% controlled using deionized water and dropwise addition of the monomer methyl methacrylate and vinyl-POSS. The stability of the prepared aqueous chloroprene latex was good, with a small amount of gel appearing at 4% and 5% content for polymerization, and the best monomer conversion at 4% vinyl-POSS content. Infrared spectroscopic analysis demonstrated that MMA and OVS were grafted onto the chloroprene latex.
(2) The OVS grafting CRL kinetic study showed that the relationship between the monomer reaction rate and reactant concentration was R_p_ = K[E]^0.15^[I]^0.30^[OVS]^1.05^ (K is a constant); the reaction mechanism deduced the rate equation R_p_ = k_p_(2k_d_/k_t1_)^0.5^[M][I_2_]^0.5^ and the free radical reaction mechanism of grafting polymerization is basically correct, but there is a deviation in the order of reaction because the actual experimental procedure deviates from the assumptions made in the theoretical derivation.
(3) As the addition of vinyl-POSS increased, the tensile strength of the chloroprene latex first increased and then decreased, and the thermal stability of the chloroprene latex increased.

## Data Availability

Data are contained within the article and supplementary materials.

## Supplementary Materials

The following supporting information can be downloaded at: https://www.mdpi.com/article/10.3390/polym16040462/s1, Figure S1: The TEM photograph of 5% OVS modified aqueous chloroprene latexes; Table S1: Physical properties of OVS modified waterborne neoprene emulsion; Table S2: Stability testing of OVS-modified neoprene emulsions; Table S3: The corresponding detailed data obtained from tensile measurements; Table S4: Thermal stability of OVS modified waterborne neoprene.
