# Supplementary material for: Preparation and Characterization of Chloroprene Latexes Modified with Vinyl-POSS
Source: Polymers (Basel). 2024 Feb 7;16(4):462. doi: 10.3390/polym16040462 (PMC10891617; doi:10.3390/polym16040462)
Supplement: Supplementary file 1 [file polymers-16-00462-s001.zip › polymers-2815438-supplementary.pdf]

Preparation and Characterization of Chloroprene Latexes Modified with Vinyl-POSS

Junhua Chen [1,2], Zhenxian Wu [1], Qingwei Wang [1], Chuanghui Yang [1], Jinlian Chen [1], He Zhang [1,2], Yinping Wu [1,2], Dong Yu Zhu [3], Xiangying Hao [1,2*]

[1] School of Environmental and Chemical Engineering, Zhaoqing University, Zhaoqing 526061, China;

[2] Guangdong Provincial Key Laboratory of Environmental Health and Land Resource, College of Environmental and Chemical Engineering, Zhaoqing University, Zhaoqing 526061, PR China;

[3] School of Chemical Engineering and Light Industry, Guangdong University of Technology, Guangzhou 510006, China

Table S1. Physical properties of OVS modified waterborne neoprene emulsion

Samples	Viscosity(Pa.s)	Conversion(%)
OVS-0%	21.00	60.29
OVS-1%	20.70	77.91
OVS-2%	19.80	79.98
OVS-3%	19.50	83.95
OVS-4%	17.70	88.71
OVS-5%	16.80	84.32

Table S2. Stability testing of OVS-modified neoprene emulsions

Samples	appearance	dilute stability	Storage at room temperature /days	high-temperature storage /days
OVS-0%	gel-free	√	＞90	＞15
OVS-1%	gel-free	√	＞90	＞15
OVS-2%	gel-free	√	＞90	＞15
OVS-3%	gel-free	√	＞90	＞15
OVS-4%	A small amount of gel	√	＞90	＞15
OVS-5%	A small amount of gel	√	＞90	＞15

(√ means "pass"; × means "fail")

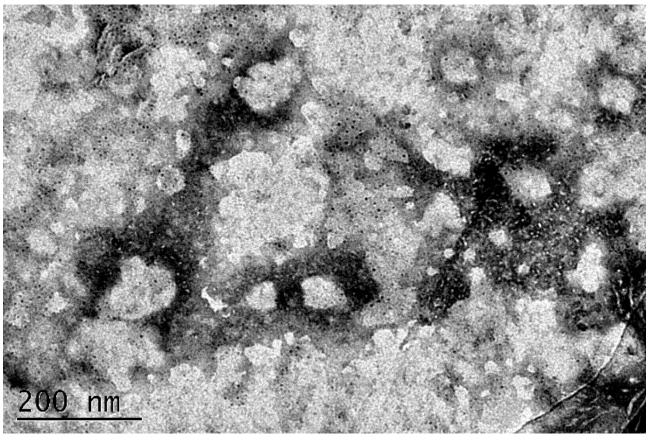

Figure S1. The TEM photograph of 5% OVS modified aqueous chloroprene latexes

Table S3 The corresponding detailed data obtained from tensile measurements.

Formulations	Tensile strength (MPa)	Breaking elongation (%)
OVS-0%	0.87	304.2
OVS-1%	1.21	423.4
OVS-5%	1.45	523.3

Table S4. Thermal stability of OVS modified waterborne neoprene

**Samples**	**T_{max1} (°C)**	**T_{max2} (°C)**	**Y_c at 600 °C (%)**
OVS-0%	377.3	445.9	18.58
OVS-5%	372.6	450.4	21.64
